# Ophthalmology

**Published:** 1895-11

**Authors:** Alvin A. Hubbell

**Affiliations:** Professor of ophthalmology and otology in the Medical Department of Niagara University


### Progress in Medical Science.

### OPHTHALMOLOGY.

By ALVIN A. HUBBELL, M. D.,

Professor of ophthalmology and otology in the Medical Department of Niagara University.

### EDEMATOUS OPTIC NEURITIS OF INTRACRANIAL ORIGIN.

DR. H. PARINAUD, of Paris, (*Annales d' Oculistique*, English edition, July, 1895,) after reviewing the several theories of the pathogenesis of optic neuritis, explains its production in the following manner : optic neuritis of intracranial origin is primarily a lymphatic edema of the nerve. This edema is produced by the same influences and the same mechanism as the edema in the cerebral substance, of which the optic nerve forms a sort of orbital prolongation. It is most frequently combined with hydrocephalus and the increase of intracranial tension which accompanies it, but it does not necessarily imply the existence of ventricular hydropsy. Furthermore, the excess of intracranial tension seems to be incapable alone of producing capillary edema.

Edema of the optic nerve does not imply a considerable excess of intracranial tension, or mechanical forcing back of the intracranial fluid into the nerve, any more than edema of the legs in cardiac or abdominal affections implies a strong elevation of venous tension, or forcing of serous fluid into the extremities.

The inter-vaginal infusion of the optic nerve is a concomitant phenomenon which has no relation of cause and effect with neuritis. The latter would occur quite as well if there were no intervaginal space.

The scleral ring favors the papillary strangulation produced by this edema in the same sense as a ligature on an edematous limb. On the other hand, the external seat of the optic nerve plays a protective rôle in relation to the nerve itself, in the same way as a compress bandage does on an edematous limb.

For these reasons he designates optic neuritis of intracranial origin by the name of edematous neuritis, which seems likely to be accepted.

---

### FORMOL IN THE TREATMENT OF PURULENT OPHTHALMIA.

Dr. Fromaget, of Bordeaux, (*Annales d'Oculistique*, English edition, February, 1895,) writing of formol in the treatment of purulent ophthalmia, reports good results. He says that formol, the antiseptic qualities of which have been accurately established, and which was first recommended in ophthalmology by Valude, he has employed in two ways: as a collyrium in the proportion of 1 to 200, and as a wash, 1 to 2000. After carefully bathing the palpebral and bulbar conjunctiva with a solution of 1 to 2,000, several drops of the collyrium should be instilled four times a day.

Formol has already been used successfully in cases of catarrhal conjunctivitis, and has also been submitted to the new-born children from the obstetrical clinic of the Hospital St. André, of which ten cases are reported without accident or injury to the cornea. When used at a later stage the results were also favorable, and where corneal lesions already existed they did not increase, although nitrate of silver and sublimate irrigations had not retarded the progress of the disease. Can it be said that the use of nitrate of silver would not have produced as good results? But to acknowledge that the results produced by formol can be compared to those of the nitrate is to affirm at the same time the value of formol.

In one case, a young girl seventeen years of age attacked with purulent ophthalmia, and in which the disease was hardly twenty-four hours old, the results obtained by the use of formol were very satisfactory. It was not successful, however, in two cases of blennorrheic ophthalmia in adults. These two patients lost their sight from complete ulceration of the cornea with perforations. These are the only two failures which he has to record. These last results are certainly not brilliant. But it is to be admitted that in spite of the most energetic treatment with nitrate of silver, eyes are not infrequently lost, and that speedily. The most serious purulent ophthalmias are those of blennorrheic origin observed in adults, while the majority of ophthalmias of the new-born are far more benign.

Formol is not altered by light, it does not rust instruments, it is antiseptic and presents but a single disadvantage, that of being painful in strong solution—for example, 1 to 200. But in proportion of 1 to 2000 it is well borne and produces no pain.

---

BISMUTH LORETINATE DRESSING IN THE TREATMENT OF EYE DISEASES.

In the treatment following operations on the eye, as well as in the treatment of traumatisms and various ocular affections, (phlyctenular, catarrhal, purulent and granular ophthalmia, diphtheria, tuberculosis, epithelioma and septic ulcers of the cornea,) Dr. W. Nicati, Marseilles, (*The Medical Week*, September 6, 1895,) has for some time successfully employed bismuth loretinate.

Dr. Nicati uses this substance both for "internal spraying" of the eye, in the same manner as calomel has long been applied, and for antiseptic dressing. The application of this dressing is described by Dr. Pietri in his thesis for graduation as follows: the patient opening his eye, powdered bismuth loretinate is sifted by means of a dry brush over the whole ocular region, including the eyebrows; then a strip of court plaster, about five centimeters in length by one in width, which has been dipped in an antiseptic solution, is applied vertically over the eye. Over this strip, an aseptic cotton-wool tampon is so arranged as to cover completely the orbital region.

The dressing is maintained by means of a bandage which may be renewed daily without inconvenience; it is even imperative to renew it whenever it has become displaced. Its therapeutical effect is most marked in cases of phlyctenular ophthalmia. Under the influence of the bismuth loretinate the pain and photophobia are said to promptly cease and rapid recovery follows. In cases of pustules, associated with ulcers, application of the dressing may be advantageously preceded by cauterisation of the diseased spots by the fine point of the galvano-cautery.

In ciliary blepharitis, Dr. Nicati obtains excellent results from applications of an ointment, prepared by mixing bismuth loretinate with a sufficient quantity of olive oil, rubbed on the eyelashes with a small wad of cotton-wool. This ointment softens the crusts and causes them to readily fall, thus favoring a cure of the palpebral affection.

---

### ON THE USE OF LARGE SUB-CONJUNCTIVAL INJECTIONS.

Dr. de Wecker, of Paris, (*Annales d' Oculistique*, English edition, June, 1895,) has returned to large sub-conjunctival injections and employs sublimate in solution of 1-2000. The therapeutic result has been so conclusive, as compared with that of weak injections, that doubt as to their rapid efficiency has not been tenable, either for him or for his pupils, who have carefully followed his work, and who have witnessed their surprising effects in a number of patients.

He ordinarily injects half a Pravaz syringeful, using the following formula:

| | | |
|---|---|---|
| Sublimate .......................... | 0.015 | gramme. |
| Salicylate of eserine .................. | 0.05 | gramme. |
| Sterilised distilled water ............... | 30.00 | grammes. |

The myotic is only replaced by atropine or scopolamine when the case is complicated with iritis, and the combination of the sublimate solution with a myotic has seemed to produce a more favorable action than the simple sublimate solution.

The injections are first performed daily, during from one to three days after preparatory disinfection of the lashes with a 1 per cent. solution of oxycyanide of mercury and irrigation of the conjunctiva with a 4 per cent. boric acid solution. In antiseptic occlusion, bandage for the eye completes the treatment. The intervals for the injections and the amount of fluid injected are regulated according to the improvement. Even in very advanced cases of suppuration of the cornea with hypopyon, only six or eight injections are necessary to obtain recovery with clearing up of the diseased portions.

In using, exclusively, solutions of 1-2000 he claims to have never seen mortification of a portion of the conjunctiva, severe pains, or edema of one or the other lid, accidents described by his colleagues as "almost constant." Far from that, the injections have seemed to be scarcely painful, if the eye was well cocainised; they have an undoubted calming action. Patients with large ulcerations of the cornea have recovered sleep, of which they had been deprived from the commencement of their disease, and almost all spoke of the great relief produced by the injections.

Occlusion of the eyes and large sub-conjunctival injections have been the only method of treatment employed in a large majority of destructive affections of the cornea, and, therefore, to them alone can the curative power be attributed. It is of slight importance whether this is due to antisepsis or to acceleration of lymph current in the cornea, or, finally, to the circulatory modifications which occur, all influences whose quantitative action it will never be possible to verify.

---

### THE USE OF CONVEX GLASSES FOR DISTANT VISION IN MYOPIA.

Drs. L. de Wecker and J. Masselon (*Annales d'Oculistique*, English edition, February, 1895,) discuss the advantage gained in certain cases of myopia from the use of convex glasses for distant vision. They say that although this seems at first paradoxical, it is unquestionably true that myopes succeed in considerably increasing their sharpness of vision and are enabled to read characters at a distance which they could not distinguish with concave glasses, such, for example, as the name of a street or the number of a house, by placing in front of the better eye a convex glass, as they would use a lorgnette. The inconvenience of this procedure is in the fact that an inverted image is obtained ; but with a little practice inverted characters are easily read, and the facts prove conclusively that this inconvenience is amply compensated for by the increased size of the image, since we know of myopic persons who make habitual use of such glasses.

The method of employing convex glasses in myopia consists in placing the glass before the eye at such a distance that its focus coincides with the punctum remotum. If the myope completely relaxes his accommodation, as he must do under other circumstances, he finds that with the aid of this convex glass the eye is adjusted for parallel rays and can see at a distance.

Practically, glasses weaker than 5 dioptries cannot be employed and it is desirable that they should be five or six centimeters in diameter. Under these conditions, the myope, with a little practice, can easily see the objects which he desires to observe, and the visual acuity will be found to be more than double that which is obtained by correcting the myopia with a concave glass, as we have recently had occasion to observe in a case of a person with myopia.

of 20 dioptries who had accustomed himself to the use of a convex lens.

It would be advantageous to extend the use of convex glasses to many cases where, with a high degree of myopia, the visual acuity is defective. Glasses of about +6 dioptries should be prescribed and used like a magnifying glass for distant vision. Since these glasses produce an inverted image, they are to be used only occasionally, when the myope wishes to see with precision small objects, such, for example, as a public notice, the name of a street, or the number of a house. In these special instances convex lenses will be regarded by certain myopic persons as quite superior to concave lenses, and they will be thankfully accepted as a valuable expedient.

---

### EYE-GLASSES.

DR. HANSELL, of Philadelphia, defends the use of eye-glasses as follows: "It will be admitted that spectacles are an unbecoming addition to anyone's face. However light, the screws in the lenses and the fastenings of the hooks as well as the hooks themselves are conspicuous and materially alter the wearer's expression. Eye-glasses are unqestionably more becoming. They are neater, more graceful, smaller and their means of support practically invisible. The advocates of the spectacle frame claim that it will not fall off, will maintain its position on the nose and, therefore, hold the lenses in their proper adjustment, is comfortable and durable and though in appearance objectionable is therapeutically to be preferred.

"On the other hand, I contend that the eye-glasses, the 'pince-nez,' offers decided advantages, and should be chosen by the large majority of those who must wear one or the other. The minority consists of those whose occupations demand severe bodily exertion, stooping position, or exposure to the weather, or who have deformities of the root of the nose.

"Spectacles indent the nose, cut the ears, are awkward to handle, are liable to be bent as they are being thrust into the case, and require constant attention and frequently the aid of the skilled mechanic to maintain accurate adjustment. The eye-glass is less conspicuous, is more convenient, particularly when far and near corrections must be worn, and will support a lens with precision equal to the spectacle frame, including cylinders and prisms, and, having a firm spring and no bars or long hooks, is far less likely to become bent and the lenses out of their proper axes or centers. I, therefore, prescribe glasses whenever in my judgment the conditions are favorable."

